# A high-efficiency method for site-directed mutagenesis of large plasmids based on large DNA fragment amplification and recombinational ligation

**DOI:** 10.1038/s41598-021-89884-z

**Published:** 2021-05-17

**Authors:** Kewei Zhang, Xiaomei Yin, Kaituo Shi, Shihua Zhang, Juan Wang, Shasha Zhao, Huan Deng, Cheng Zhang, Zihui Wu, Yuan Li, Xiangyu Zhou, Wensheng Deng

**Affiliations:** 1 College of Life Science and Health, Wuhan University of Science and Technology, Wuhan, 430065 China; 2 College of Materials and Metallurgy, Wuhan University of Science and Technology, Wuhan, 430081 China

**Keywords:** Biological techniques, Biotechnology, Molecular biology

## Abstract

Site-directed mutagenesis for large plasmids is a difficult task that cannot easily be solved by the conventional methods used in many laboratories. In this study, we developed an effective method for Site-directed Mutagenesis for Large Plasmids (SMLP) based on a PCR technique. The SMLP method combines several effective approaches, including a high-efficiency DNA polymerase for the large DNA amplification, two independent PCR reactions and a fast recombinational ligation. Using this method, we have achieved a variety of mutants for the *filamin A* gene (7.9 kb) cloned in the pcDNA (5.4 kb) or the pLV-U6-CMV-EGFP (9.4 kb) plasmids, indicating that this method can be applied to site-directed mutagenesis for the plasmids up to 17.3 kb. We show that the SMLP method has a greater advantage than the conventional methods tested in this study, and this method can be applied to substitution, deletion, and insertion mutations for both large and small plasmids as well as the assembly of three fragments from PCR reactions. Altogether, the SMLP method is simple, effective, and beneficial to the laboratories that require completing the mutagenesis of large plasmids.

## Introduction

**Figure 1:**
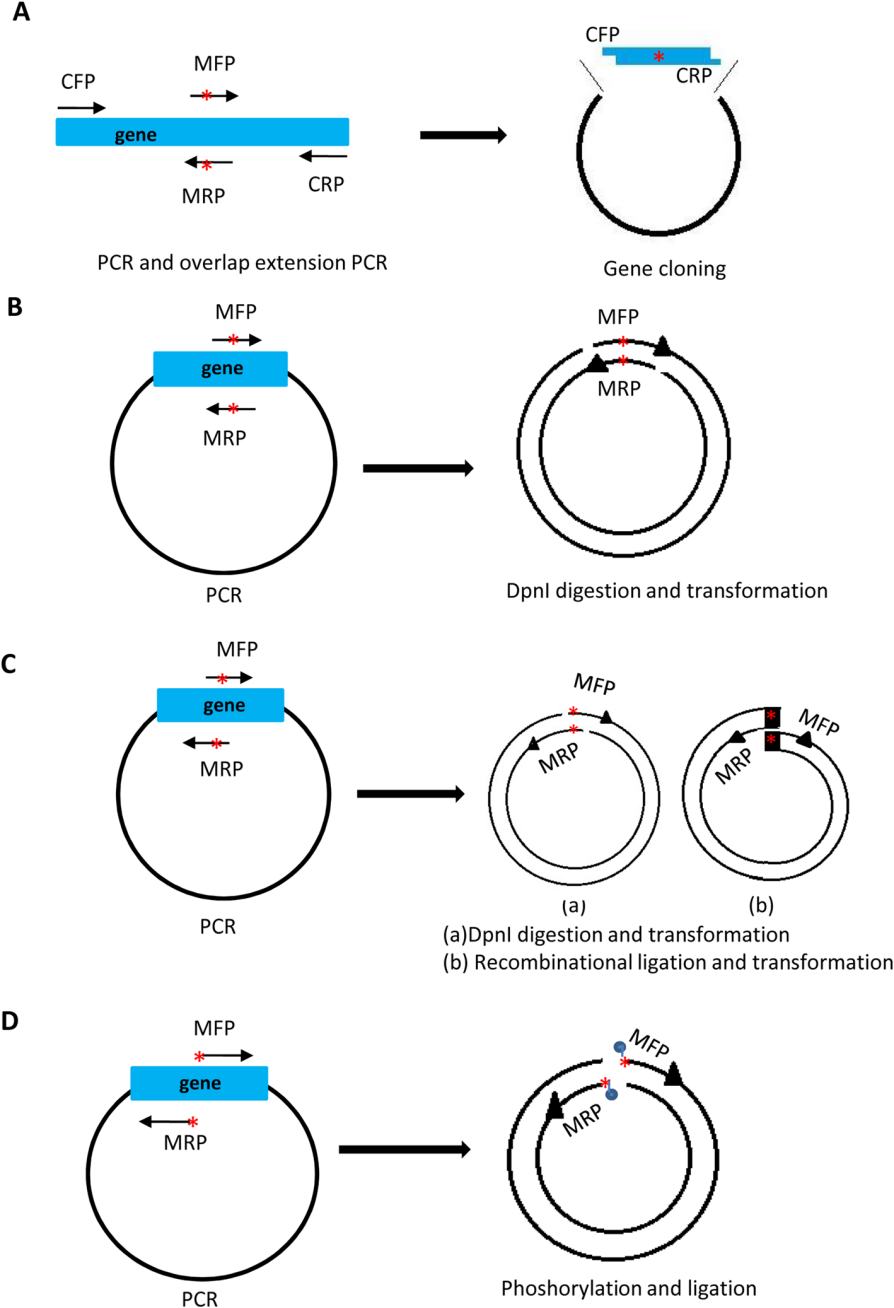
A summary showing the methods established for PCR-based site-directed mutagenesis. (**A**) A diagram for site-directed mutagenesis based on PCR, overlap extension PCR, and gene cloning. In this method, two PCR reactions are first performed with the primer pair of CFP and MRP and the primer pair of MFP and CRP. PCR products are purified and the resulting PCR products were used for overlap extension PCR with the primer pair of CFP and CRP. The products from overlap extension PCR were digested with restriction enzymes, and the resulting fragments were ligated with the plasmids digested with the same restriction enzymes. CFP, cloning forward primer; CRP, cloning reverse primer; MFP, mutation forward primer; MRP, mutation reverse primer. The red stars in the left and right panels represent mutated bases. (**B**) A diagram for site-directed mutagenesis based on PCR with a pair of complementary primers (double-strand DNA fragments), Dpn I digestion, and transformation. (**C**) A diagram for site-directed mutagenesis based on PCR with a pair of partially complementary primers, Dpn I digestion and transformation (a) or based on PCR with a pair of partially complementary DNA fragments, recombinational ligation, and transformation (b). (**D**) A diagram for site-directed mutagenesis based on PCR with a pair of inverse primers, phosphorylation, ligation, and transformation. The blue dots in the left panel represent phosphorylated groups. The red stars in A-D represent mutated bases.

Site-directed mutagenesis, one of the most important techniques in molecular biology, has widely been used to investigate the structures and functions of nucleic acids and proteins, the mechanisms of genetic diseases, and the effect of genome modification^1–4^. Although gene mutations, including point substitution, deletion, and insertion, can be achieved by a variety of methods^5,6^; site-directed mutagenesis mediated by polymerase chain reaction (PCR) is one of the most powerful approaches to generate gene mutants in vitro. So far, many PCR-based methods for site-directed mutagenesis have been developed^5,6^; among which, site-directed mutagenesis by overlap extension PCR has first been developed^7,8^. In this method two independent PCR reactions are performed, the resulting PCR products are used for an overlap extension PCR reaction. The products from the overlap extension PCR reaction are eventually cloned into plasmids. However, the drawbacks of this method are that the overlap extension reaction fails frequently, and the method is labour-consuming. One of the simplest methods for site-directed mutagenesis is the method based on PCR with a pair of complementary primers (a double-stranded DNA fragment), where the plasmids acting as DNA templates during PCR are required to be digested by the restriction enzyme *Dpn*I before transformation^9,10^. This method has widely been applied to the generation of gene mutants in many laboratories. For instance, the Quick-Change Site-Directed Mutagenesis Kit (Thermo Scientific), one of the site-directed mutagenesis kits mostly used, has been developed based on this method. However, the efficiency of PCR reaction in this method is extremely low due to the formation of primer-dimers, which often leads to the failure of mutagenesis. To overcome this drawback, scientists have developed an improved method using a pair of partially complementary primers in PCR reaction^6,11^. Although the partially complementary primers reduce the chance to form the primer dimers during PCR, PCR products with homologous ends are possibly produced in the reaction (see the summary in Fig. 1C). On this occasion, the gene mutants cannot be obtained by directly transforming PCR products into bacteria after *Dpn* I digestion unless the PCR products are purified and subjected to recombinational ligation and transformation.

An effective method to avoid the formation of the primer dimers is that PCR for site-directed mutagenesis is performed using a pair of inverse primers^12,13^. This method has been confirmed to be effective for mutagenesis of small plasmids^12,13^. However, this method is time-consuming because PCR products have to be purified, phosphorylated, and ligated before the transformation. Besides, the efficiency of DNA phosphorylation is usually so low that site-directed mutagenesis does not succeed at times even if PCR reactions are effective. Apart from the methods described above, Edelheit and colleagues have reported a PCR-mediated method for site-directed mutagenesis, where two independent PCR reactions are performed using either forward or reverse single primers. The resulting PCR products from each reaction are combined at 95℃, followed by annealing at 37℃, digestion with *Dpn* I, and transformation with bacterial cells^14^. The disadvantage of this method perhaps is yet the low efficiency for mutagenesis due to single primers used in PCR reactions. Previous studies have shown that site-directed mutagenesis can be completed by combining PCR with DNA recombination techniques^15–18^*.* Kitagawa and Abdulle have reported a method for site-directed mutagenesis in vivo using three fragments from PCR and a homologous recombination system^15^. Recently, Trehan and colleagues have established a one-step method for site-directed mutagenesis using a bacterial system expressing a viral recombination protein and the products derived from PCR with a pair of partially complementary primers^18^. However, the bacterial system has to be prepared before mutagenesis.

Despite the availability of numerous methods for site-directed mutagenesis, mutagenesis of large plasmids remains difficult in vitro. In this study, we developed an effective method that can be applied to site-directed mutagenesis of large plasmids (SMLP). We show that the SMLP method is highly efficient for the mutagenesis of the plasmids up to 17.3 kb and can be utilized to generate substitution, deletion, and insertion mutations for both large and small plasmids.

## Results

### The conventional methods for site-directed mutagenesis are difficult to generate mutants for large plasmids

PCR-based methods for site-directed mutagenesis have been playing an important role in the generation of gene mutants in vitro. In our recent work, we tried to generate several mutants for the *filamin A* gene (*FLNA*, 7.94 kb) cloned into the pcDNA 3.1 ( +) plasmid (5.4 kb) using the conventional methods as indicated in Fig. 1 and the primers required for each method. However, we couldn’t gain any mutant through these methods although different primer pairs were tried in those PCR reactions (Table S1 and Table S3). We supposed that the reason for failure in the mutagenesis of 13.3 kb plasmid was due to either the unsuccessfulness of overlap extension PCR for large DNA fragments (Fig. 1A) or the abortive PCR for large plasmids (Fig. 1B–D). Another reason is perhaps that the *Pfu* DNA polymerase (Promega) used in these assays was unable to amplify big-sized plasmids. The Q5 high fidelity DNA polymerase (New England Biolabs) has been confirmed to generate gene mutants from the plasmids up to 12 kb^19^. Thus, we replaced the *Pfu* DNA polymerase in the original systems with the Q5 high fidelity DNA polymerase (NEB) and performed site-directed mutagenesis using their respective methods shown in Fig. 1 and the primers included in Table S1 and Table S3. However, no mutant was obtained by these methods although the Q5 high fidelity DNA polymerase was utilized in PCR reactions.

### Establishment of the site-directed mutagenesis of large plasmids based on PCR

**Figure 2:**
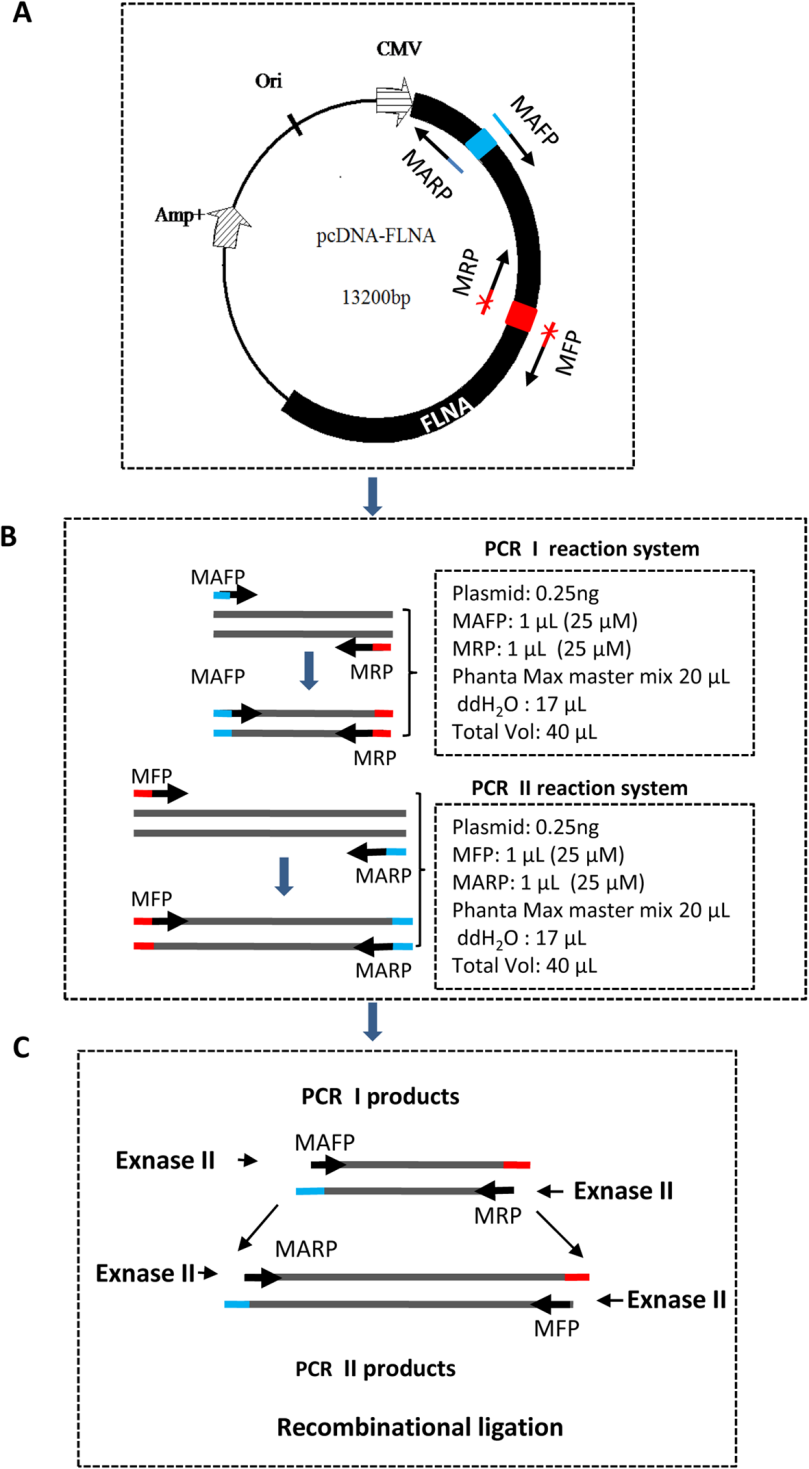
Establishment of the method for site-directed mutagenesis of large plasmids (SMLP) based on PCR. (**A**) A plasmid map showing the locations of the PCR primers designed. The pcDNA-FLNA plasmid is presented in the diagram, and two pairs of PCR primers are designed as indicated. MFP represents a mutation forward primer; MRP represents a mutation reverse primer; MAFP is a mutation-assisting forward primer; MARP is a mutation-assisting reverse primer. The blue or red colours represent partially complementary (overlap) regions between two primers. (**B**) A diagram showing two independent PCR reactions, PCR I and PCR II. PCR I is performed using the primer pair of MAFP and MPR; whereas PCR II is performed using the primer pair of MFP and MARP. The PCR I and II reaction systems were presented on the right of the panel. (**C**) A diagram showing the recombinational ligation of the products from the PCR I and PCR II under the Exnase II. The short black arrows represent the places digested by Exnase II; the long black arrows show how two DNA fragments are ligated.

To solve the problem of **s**ite-directed mutagenesis for large plasmids, we changed the strategy for PCR-mediated mutagenesis. In the new method, one PCR reaction for large plasmids was divided into two independent PCR reactions (PCR I and PCR II). PCR primers were designed based on the following guidelines: (1) Two pairs of partially complementary primers are designed and each pair of primers were separated and used in two independent PCR reactions. (2) One pair of primers acting as mutation-assisting primers (MAFP and MARP) are designed at any known sequence in the vector and located either upstream or downstream of a mutation site (see Figs. 2A and 9A). Another pair of primers, mutation primers (MFP and MRP), are designed according to experimental purposes. (3) The distance between two pairs of primers may vary from 60 bp to 15 kb (Fig. S1). (4) The length of each primer should be 33–35 bp where the point mutation site is usually designed in the overlap region (Fig. S1). (5) The overlap region for each pair of primers is 15–20 bp, and the non-overlap region for each primer is 18–20 bp (Fig. S1). (6) The content of GC bases for each primer is between 45 and 65%, and the primer should avoid over 4 consecutive G or other bases.

As described above, each pair of partially complementary primers were separated and used in PCR I and PCR II, where 1 μL of 25 μM forward and reverse primers was used in the PCR reaction (Fig. 2B). PCR I was performed using the primer pair of MAFP and MRP, whereas PCR II using the primer pair of MARP and MFP (Fig. 2B). To ensure PCR I and PCR II reactions successful, we performed PCR using the Phanta Max master mix (Vazyme Co.) that contains the super fidelity DNA polymerase amplifying a DNA fragment up to 20 kb (Fig. 2B). To generate the mutants for large plasmids, we purified the DNA obtained from PCR I and PCR II using the gel extraction kit (CWBiol, China). The resulting DNA fragments were mixed at the ratio of 1:1 in moles where the amount of DNA for any of the fragments was not less than 30 ng. Recombinational ligation was performed using an Exnase II kit as described in the section of the Methods (Fig. 2C). Three μL ligation samples were used for transformation, and all of the transformation mixtures were spread in one LB plate. The colonies on the LB plates were selected, inoculated, and cultured overnight for plasmid preparation. The resulting plasmids were detected by agarose gel electrophoresis, and the plasmids with the correct size were verified by DNA sequencing. From now on, we designated the method for site-directed mutagenesis of large plasmids as the SMLP method.

**Figure 3:**
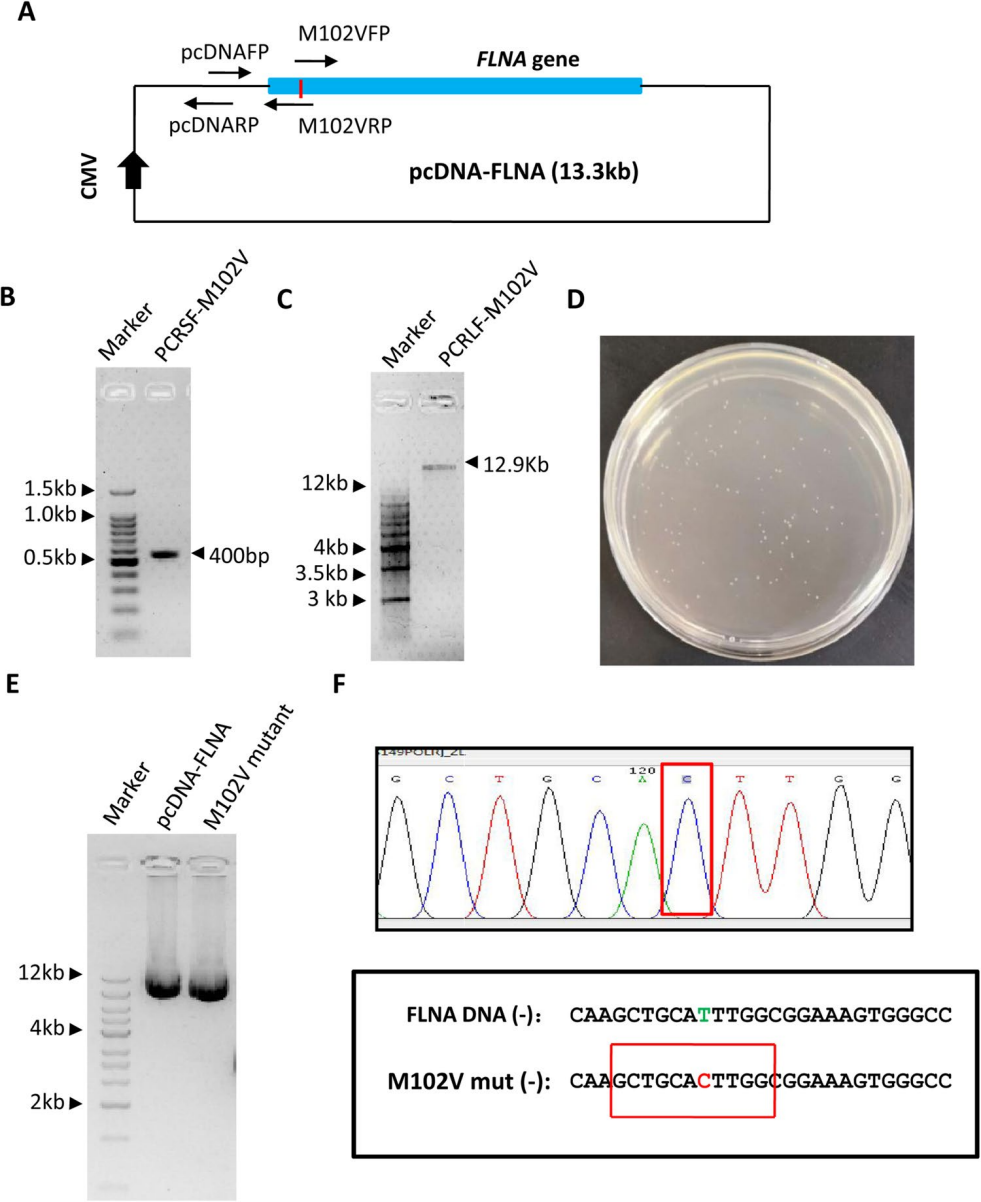
The SMLP method can be used for mutagenesis of the plasmid about 13.3 kb. (**A**) A diagram showing the pcDNA-FLNA plasmid and the primers designed for sit-directed mutagenesis. pcDNAFP represents a pcDNA plasmid forward primer; pcDNARP represents a pcDNA plasmid reverse primer. M102VFP is an M102V forward primer; M102VRP is an M102V reverse primer. The red line represents the mutated site in the plasmid. (**B**) Analysis of the small DNA fragments from PCR by agarose gel electrophoresis. PCR was performed using the pcDNA-FLNA plasmid as DNA templates, the primer pair of pcDNAFP and M102VRP, and Phanta Max Super-fidelity DNA polymerase. PCRSF-M102V represents the PCR small fragments for the mutagenesis of M102V. (**C**) Analysis of the large DNA fragments from PCR by agarose gel electrophoresis. PCR was performed as in (**B**) using the primer pair of pcDNARP and M102VFP. PCRLF-M102V represents the PCR large fragments for the mutagenesis of M102V. (**D**) The result of the transformation with 3μL ligation samples for the mutagenesis of M102V. The transformation plate was pictured using an EOS digital camera (Canon). (**E**) Detection of the plasmids from the mutagenesis of M102V by agarose gel electrophoresis. DNA plasmids were prepared using a Miniprep kit (CWBiol, China), and were subjected to detection with agarose gel electrophoresis. (**F**) The result of DNA sequencing for the M102V mutant. The DNA sequencing map for the M102V mutant was presented in the upper panel in which a mutated base was framed by the red rectangle lines. Both original and mutated DNA fragments were presented in the bottom panel, where the bases displayed in the sequencing map were outlined by the red rectangle lines. The original and mutated bases were presented in green and red colours, respectively.

To test whether the SMLP method can be used for the generation of mutants for large plasmids, we initially generated a mutant (M102V) for the pcDNA-FLNA plasmid (13.3 kb) using the primers as indicated in Fig. 3A and Table S2. Two independent PCR reactions were carried out as described in Fig. 2B. Agarose gel electrophoresis showed that both small and large fragment products were obtained from PCR I and PCR II, respectively (Fig. 3B,C). PCR products were purified and were then subjected to recombinational ligation. Three μL ligation samples were transformed with *E. Coli* competent cells, all of the transformation mixtures was spread in one LB plate containing ampicillin with the final concentration of 50 μg/mL. As expected, around one hundred and fifteen colonies were observed from one LB plate (Fig. 3D). Next, we inoculated 5 mL LB medium by picking a single colony from the transformation plate; plasmids were prepared and detected by agarose gel electrophoresis. Figure 3E shows that the M102V mutant has the same size as the original plasmids (pcDNA-FLNA) although they appeared in a super-coiled form. Since PCR products were purified by DNA gel extraction kit before ligation, the pcDNA-FLNA plasmid with the correct size was likely mutated. Indeed, DNA sequencing confirmed that the M102V mutant of the pcDNA-FLNA plasmid has been achieved (Fig. 3F). These data indicate that the SMLP method can be applied to generating gene mutants for large plasmids.

### The SMLP method produces consistent results in the mutagenesis of large plasmids

**Figure 4:**
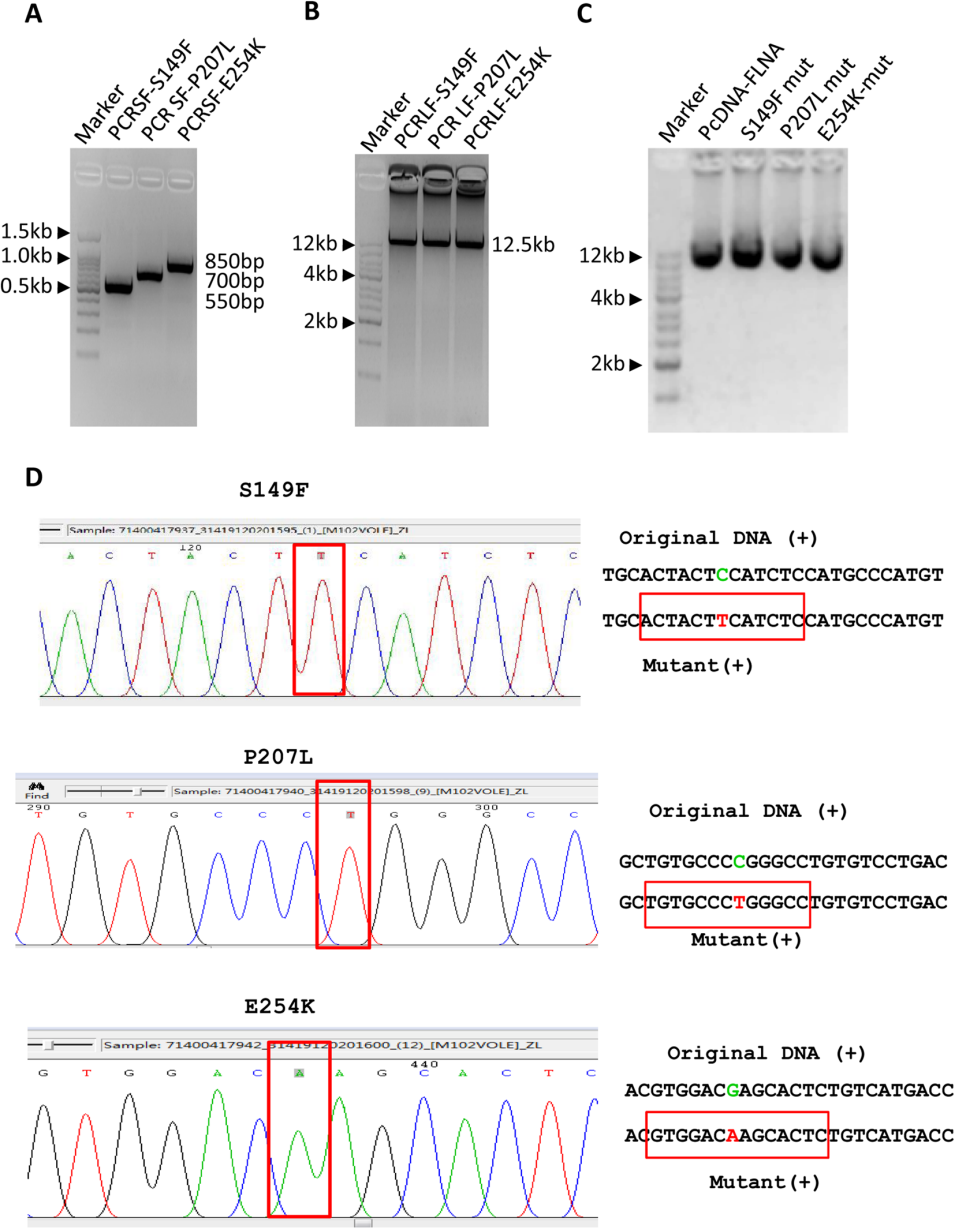
The SMLP method showed consistent results in the mutagenesis of large plasmids. (**A**) Analysis of the small DNA fragments from PCR by agarose gel electrophoresis. PCR was performed using the primer pcDNAFP and one of the mutation reverse primers, including S149FRP, P207LRP, and E254KRP, where the pcDNA-FLNA plasmids acted as DNA templates. PCR products were monitored by agarose gel electrophoresis. PCRSF represents PCR small fragments. (**B**) Analysis of the large DNA fragments from PCR by agarose gel electrophoresis. PCR was performed using the primer pcDNARP and one of the mutated forward primers, including S149FFP, P207LFP, and E254KFP, where the pcDNA-FLNA plasmids acted as DNA templates. PCR products were monitored by agarose gel electrophoresis. PCRLF represents PCR large fragments. (**C**) Analysis of the pcDNA-FLNA plasmids from site-directed mutagenesis by agarose gel electrophoresis. The plasmids from mutagenesis were prepared and analysed by agarose gel electrophoresis. (**D**) The results of DNA sequencing for S149F, P207L, and E254K mutants. The DNA sequencing maps for the S149F, P207L, and E254K mutants were presented at the left, where the mutated bases were outlined by the red rectangle lines. The right panels are the original and mutated DNA sequences for each mutant, where the bases in the sequencing map were framed by the red rectangle lines. The original and mutated bases were displayed in green and red colours, respectively.

**Figure 5:**
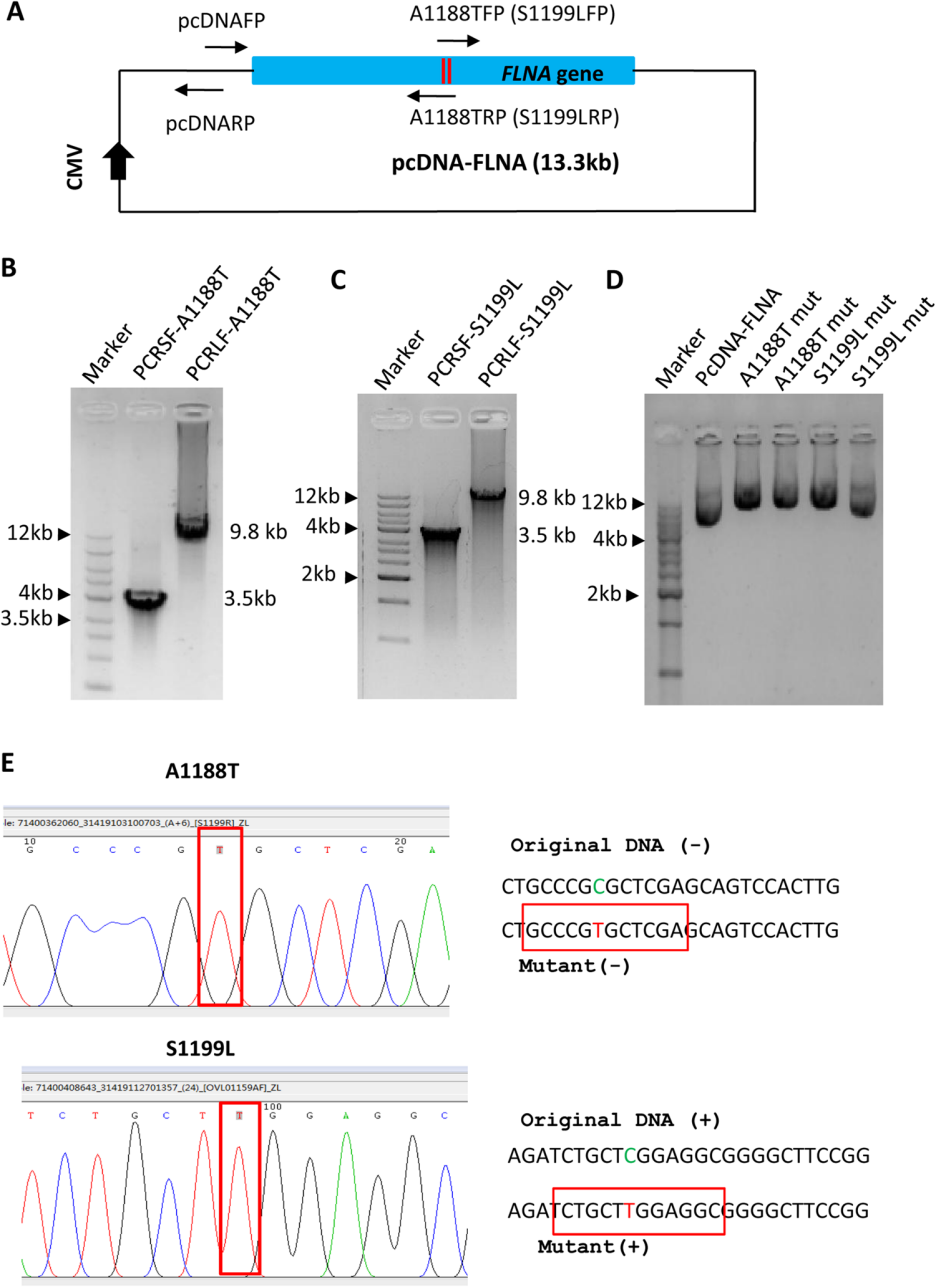
Alteration of the distance between two pairs of primers does not affect the mutagenesis efficacy of large plasmids. (**A**) A diagram showing the pcDNA-FLNA plasmid and the positions of the PCR primers designed. A1188TFP (or S1199LFP), A1188T (or S1199L) forward primer; A1188TRP (or S1199LRP), A1188T (or S1199L) reverse primer. (**B**) Analysis of the small DNA fragments from PCR by agarose gel electrophoresis. PCR was performed using the primer pair of pcDNAFP and A1188TRP or the primer pair of pcDNAFP and S1199LRP. PCR products were detected by agarose gel electrophoresis. PCRSF represents PCR small fragments. (**C**) Analysis of the large DNA fragments from PCR by agarose gel electrophoresis. PCR was performed using the primer pair of pcDNARP and A1188TFP or the primer pair of pcDNARP and S1199LFP. PCR products were detected as for (**B**). PCRLF represents PCR large fragments. (**D**) Analysis of the plasmids from site-directed mutagenesis by agarose gel electrophoresis. The plasmids derived from site-directed mutagenesis with the SMLP method were directly detected by agarose gel electrophoresis after plasmid preparation. These plasmids displayed in a super-coiled form (S1199L in the last lane) or in a linear form (the 3rd-5th lanes). (**E**) The results of DNA sequencing for the A1188T and S1199L mutants. The left panels are DNA sequencing maps for the A1188T and S1199L mutants, where the mutated bases are framed by the red rectangle lines. The right panels are the original and mutated DNA fragments for each mutant presented as for Fig. 4D.

To determine whether the result of mutagenesis for large plasmids is reproducible, we tried to generate different mutants for the pcDNA-FLNA plasmid using the same method as for the generation of M102V mutant. PCR was performed using the pcDNA-FLNA plasmids as DNA templates and the primers included in Table S2. Agarose gel electrophores showed that both small and large DNA fragments were obtained from PCR reactions (Fig. 4A,B). Plasmid detection and DNA sequencing confirmed that three mutants, including S149F, P207L, and E254K, were obtained by the SMLP method (Fig. 4C,D). These data indicate that the SMLP method can give rise to consistent results in site-directed mutagenesis of large plasmids. To substantiate this observation further, we tested the effect of the SMLP method on mutagenesis of large plasmids by increasing the distance between two pairs of primers used for PCR, where the small fragment was increased to 3.5 kb from 850 bp (Figs. 4A and 5A). We sought to generate the A118T and S119L mutants from the pcDNA-FLNA plasmids using the SMLP method. Agarose gel electrophoresis confirmed that both small and large DNA products were obtained by PCR (Fig. 5B,C), indicating that alteration of the distance between two pairs of primers does not affect PCR efficiency. Both plasmid detection and DNA sequencing confirmed that the A1188T and S1199L mutants can be achieved by the SMLP method (Fig. 5D and E). Altogether, the SMLP method can produce consistent results during the mutagenesis of large plasmids although the distance between two pairs of primers has been changed.

### The SMLP method can be applied to the generation of plasmid mutants up to 17.3 kb

**Figure 6:**
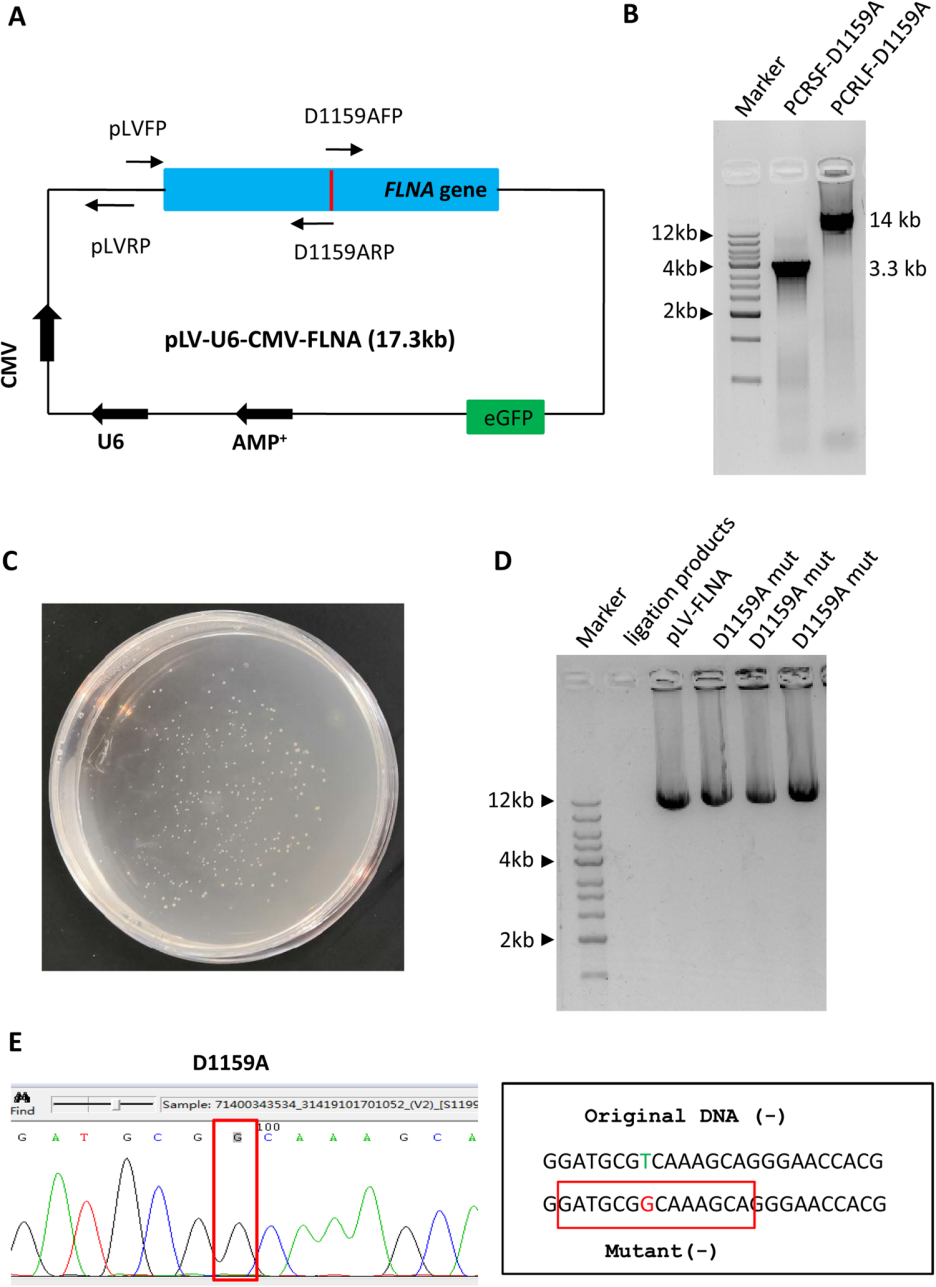
The SMLP method can be used for mutagenesis of the plasmids up to 17.3 kb. (**A**) A diagram showing the pLV-FLNA plasmid and the positions of the PCR primers designed. The primers were included in Table S2. (**B**) Analysis of the small and large DNA fragments from PCR by agarose gel electrophoresis. PCR I and PCR II were performed using the primer pair of pLVFP and D1159ARP and the primer pair of pLVRP and D1159AFP, respectively, where the pLV-FLNA plasmids acted as DNA templates. PCR products were detected by agarose gel electrophoresis as for Fig. 5B. (**C**) An image showing the result of transformation. A transformation assay was performed as described in Fig. 3D. (**D**) Analysis of the plasmids from site-directed mutagenesis by agarose gel electrophoresis. Plasmid detection was performed as for Fig. 3E. (**E**) The result of DNA sequencing for the pLV-FLNA-D1159A mutant. The left panel is the DNA sequencing map of the pLV-FLNA-D1159A mutant, where the mutated base is framed by the red rectangle lines. The right panel is the original and mutated DNA fragments presented as for Fig. 5E.

So far we have generated several mutants from the 13.3-kb plasmid using the SMLP method. Since the Phanta Max super-fidelity DNA polymerase (Vazyme Co.) can amplify large DNA up to 20 kb, we next used the plasmids with 17.3-kb in size as DNA templates and tested the efficacy of the SMLP method on site-directed mutagenesis. We constructed a pLV-U6-CMV-FLNA plasmid (about 17.3 kb) by transferring the *Flna* gene from the pcDNA-FLNA plasmid to the pLV-U6-CMV plasmid. We tried to generate a D1159A mutant for the pLV-U6-CMV-*FLNA* plasmid using the SMLP method and the primers shown in Fig. 6A and Table S2. Strikingly, both small and large fragments were amplified successfully by PCR I and PCR II even though the plasmid templates were increased to 17.3 kb (Fig. 6B and C). The PCR products were then purified, and recombinational ligation and transformation were performed as done for the generation of the M102V mutant. Figure 6D shows that about 240 colonies were observed from the LB plate, which was derived from the transformation with 3 μL ligation samples. Plasmid detection and DNA sequencing confirmed the size and sequence of the D1159A mutant correct, indicating that the SMLP method can be applied to generating the mutants from the plasmids up to 17.3 kb.

### The SMLP method is highly efficient and has a great advantage over the conventional methods mostly used in the laboratory

**Figure 7:**
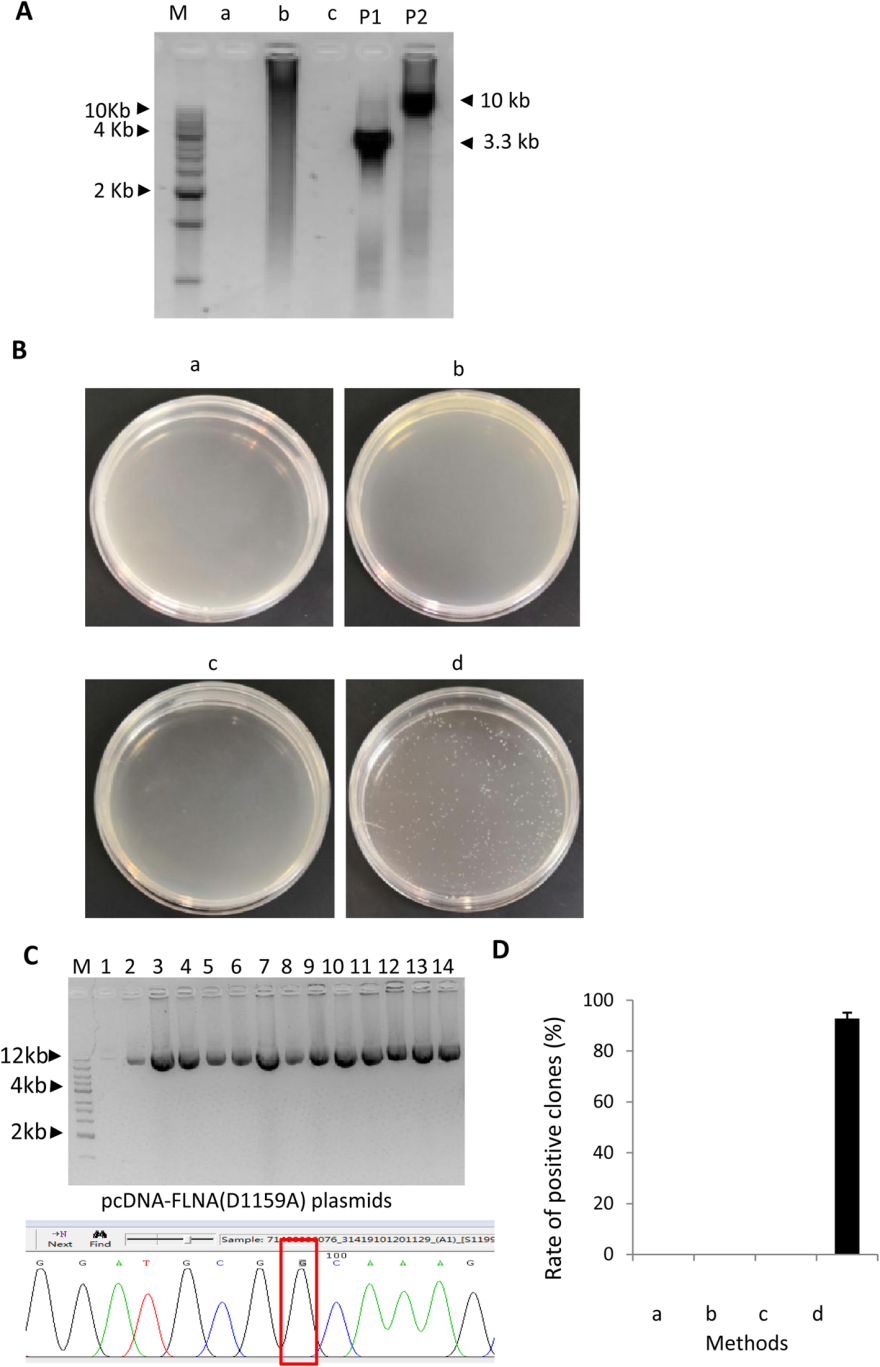
The SMLP method has an advantage over the conventional methods in the mutagenesis of large plasmids. (**A**) Detection of the PCR products from different methods for site-directed mutagenesis by agarose gel electrophoresis. a, PCR with a double-strand DNA fragment; b, PCR with a pair of partially complementary primers; c, PCR with a pair of inverse primers; P1, the small DNA fragments from PCR with the primer pair of pcDNAFP and D1159ARP; P2, the large DNA fragments from PCR with the primer pair of pcDNARP and D1159AFP. (**B**) Comparison of the transformation efficiency for the different methods for site-directed mutagenesis. The samples used for transformation were generated according to their respective methods as indicated. a, the transformation result from the method based on the PCR with a pair of complementary primers; b, the transformation result from the method based on the PCR with a pair of partially complementary primers; c, the transformation result from the method based on the PCR with a pair of inverse primers; d, the result from the SMLP method. (**C**) Analysis of the pcDNA-FLNA plasmid derived from site-directed mutagenesis by the SMLP method using agarose gel electrophoresis and DNA sequencing. Lane 1 to 13 in the image represents the plasmids from different colonies generated by the SMLP method, whereas lane 14 represents the pcDNA-FLNA original plasmid (Top panel). The mutated base in the sequencing map (bottom panel) was enclosed with the red rectangle lines as in Fig. 6E. (**D**) Comparison of the rate of positive clones from different methods for site-directed mutagenesis. a, b, c, and d in the graph represent the results from their respective methods as described in (**B**). The rate of the positive clones was obtained by calculating the percentage for the number of the mutants in the number of colonies inoculated.

To assess the efficiency of mutagenesis for large plasmids by the SMLP method, we tried to generate a D1159A mutant for the pcDNA-FLNA plasmid using the primer pairs shown in Table S3. In the meantime, three conventional methods described in Fig. 1B-D acted as controls, where the experiments for site-directed mutagenesis were performed according to their respective protocols (Fig. 1B-D) using the primer pairs included in Table S3. The Phanta Max master mix was utilized in all methods for site-directed mutagenesis. PCR products from each method were monitored by agarose gel electrophoresis. Figure 7A shows that the SMLP method only produced specific products (P1 and P2 in Fig. 7A), whereas no specific band from the other three methods was observed in an agarose gel after electrophoresis (a-c in Fig. 7A). The PCR-based methods for site-directed mutagenesis such as the methods shown in Fig. 1B and C can get success even if no product is observed in agarose gel; thus, transformation experiments were still carried on according to their respective protocols. Transformation results showed that the SMLP method gave rise to around 300 colonies when 3 μL ligation samples were transformed and spread on one LB plate. In contrast, other conventional methods didn’t produce any colony (Fig. 7B). Besides, no colony was obtained from these conventional methods even if the mutation primers were replaced by the primers designed for the generation of the A1188T and S1199L mutants (Table S3). Thirty colonies from the LB plate were inoculated with LB medium and cultured at 37 ℃ overnight for plasmid preparation. Plasmids were detected by agarose gel electrophoresis. The result showed that the rate of the plasmids with a correct size reached 93%. The plasmids with the correct size were verified by DNA sequencing, and all of the plasmids sequenced contained the D1159A site. These data indicate that the rate of the positive plasmid, in the end, can reach 93% when the pcDNA-FLNA plasmids were used for the generation of the D1159A mutant through the SMLP method. In contrast, the other three conventional methods could not give any positive plasmid. These data indicate that the SMLP method has very high efficiency in site-directed mutagenesis in vitro and a greater advantage than the conventional methods tested in this study.

### The SMLP can be used for the generation of substitution, deletion and insertion mutations for both large and small plasmids

**Figure 8:**
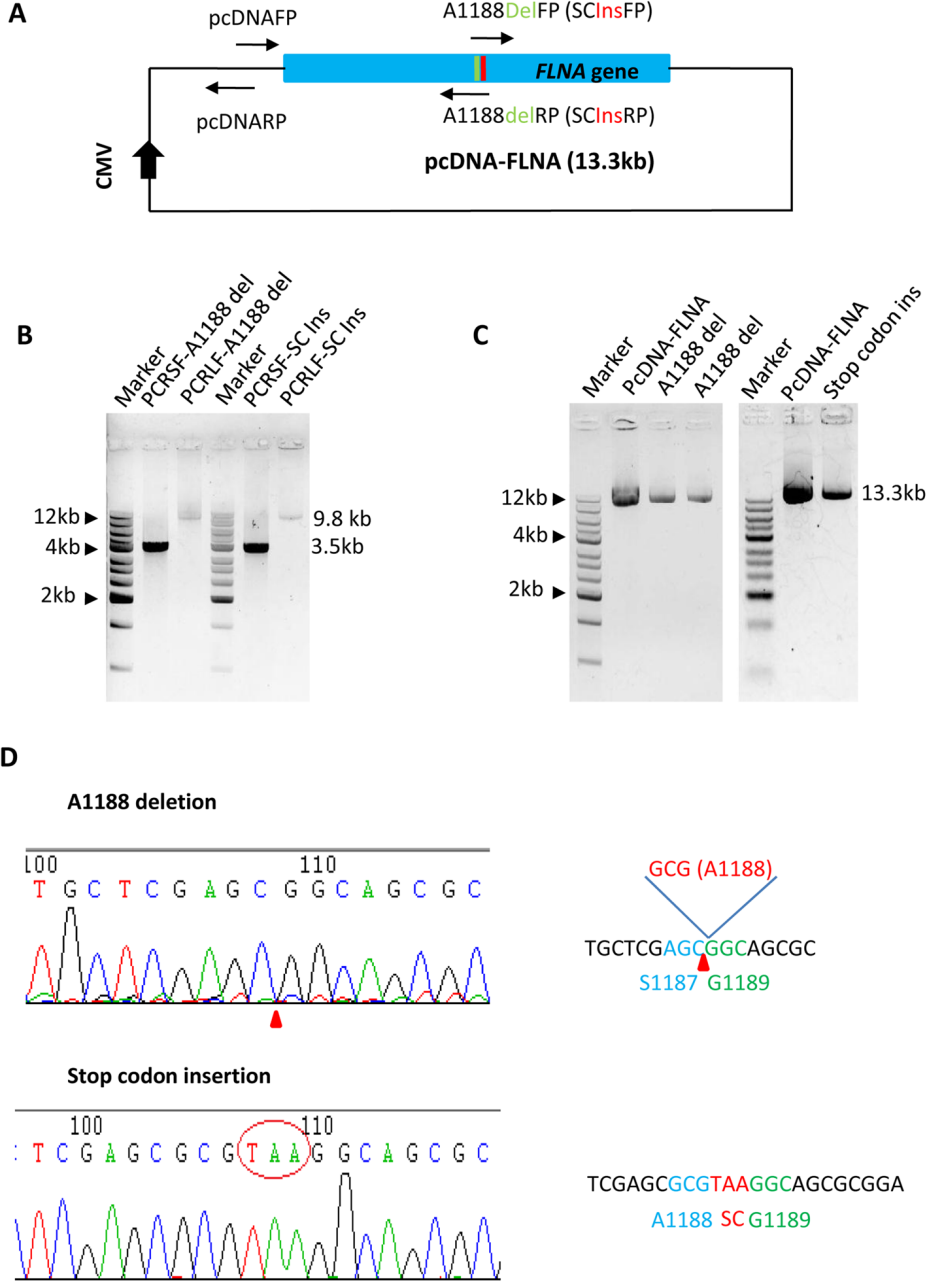
The SMLP method can be applied to the mutagenesis of deletion and insertion for large plasmids. (**A**) A diagram showing the pcDNA-FLNA plasmid and the positions of the PCR primers designed for deletion and insertion mutations. The green colour represents in the map the deletion site, and the red colour in the map represents the insert site. (**B**) Detection of the small and large DNA fragments from PCR for deletion and insert mutagenesis by agarose gel electrophoresis. PCR for small fragments was performed using the pcDNAFP primer and one of the mutation reverse primers, including A1188delRP and SCInsRP. PCR for large fragments was performed using the pcDNARP primer and one of the mutation forward primers, including A1188delFP and SCInsFP. PCR products were monitored by agarose gel electrophoresis. A1188delFP, A1188 deletion forward primer; A1188delRP, A1188 deletion reverse primer; SCInsFP, stop codon insertion forward primer; SCInsRP, stop codon insertion reverse primer. (**C**) Detection of the plasmids from deletion or insertion mutagenesis by agarose gel electrophoresis. The plasmids prepared by a Miniprep kit were directly monitored by agarose gel electrophoresis. The left panel is the detection of the plasmids from the mutagenesis of A1188 deletion, whereas the right panel is the detection of the plasmid from the mutagenesis of 1188 + TAA insertion. (**D**) The results of DNA sequencing for the mutants containing *Flna* gene deletion or insertion mutation. The left panels are the DNA sequencing maps for the mutants containing the *Flna* gene deletion or insertion. The deletion position is marked with a red triangle (top panel), whereas the insertion bases were enclosed by the red circle (bottom panel). The right panels are the DNA fragments from the sequence map where the amino acids surrounding each mutation site are indicated. SC represents a stop codon.

We have demonstrated that the SMLP method can be applied to generating the point substitution mutations for large plasmids, whether this method is suitable for the generation of deletion and insertion mutations for large plasmids was unclear. We designed and synthesized the primer pairs for the generation of an A1188 deletion mutant and an insertion mutant (A1188 + TAA) for the *Flna* gene (Table S4). PCR was performed using the pcDNA-FLNA plasmids as DNA templates and the primers synthesized above. Agarose gel electrophoresis showed that both small and large DNA fragments used for deletion or insertion mutation were obtained by PCR (Fig. 8A and B). DNA purification, ligation, and transformation were performed as for the generation of point mutations (Figs. 3, 4, 5, 6). The plasmids were prepared and detected by agarose gel electrophoresis. Figure 8C shows that the sizes of the mutants are similar to that of the original plasmid. DNA sequencing confirmed the pcDNA-FLNA mutants, including deletion and insertion, were achieved by the SMLP method (Fig. 8D).

**Figure 9:**
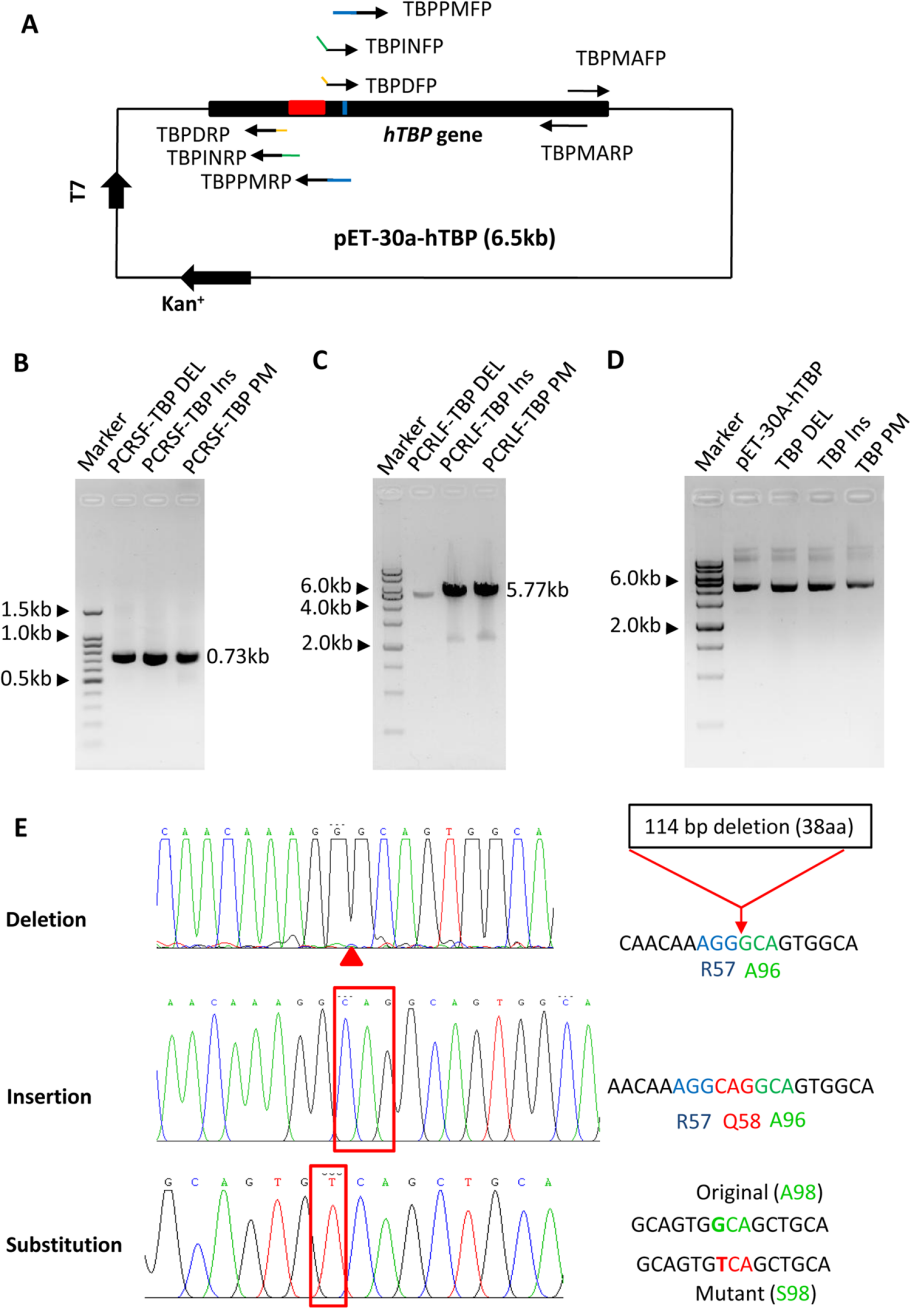
The SMLP method can be applied to the mutagenesis of deletion, insertion and point substitution for small plasmids. (**A**) A diagram showing the pET-30a( +)-hTBP plasmid and the positions of the PCR primers designed for deletion, insertion, and substitution mutations. The red region represents a deletion fragment, the blue short line represents a point mutation site, and the green short line represents the insert bases. (**B**) Analysis of the small DNA fragments from PCR by agarose gel electrophoresis. PCR was performed using the primer TBPMARP and one of the mutation forward primers, including TBPDFP, TBPINFP, and TBPPMFP. PCR products were detected by agarose gel electrophoresis. TBPMARP represents a TBP mutation-assisting reverse primer; TBPDFP, TBP deletion forward primer; TBPINFP, TBP insertion forward primer; TBPPMFP, TBP point mutation forward primer. (**C**) Analysis of the large DNA fragments from PCR by agarose gel electrophoresis. PCR was performed using the primer TBPMAFP and one of the mutation reverse primers, including TBPDRP, TBPINRP, and TBPPMRP. PCR products were detected by agarose gel electrophoresis. TBPMAFP represents a TBP mutation-assisting forward primer; TBPDRP, TBP deletion reverse primer; TBPINRP, TBP insertion reverse primer; TBPPMRP, TBP point mutation reverse primer. (**D**) Analysis of the pET-hTBP plasmids from mutagenesis by the SMLP method using agarose gel electrophoresis. The plasmids from mutagenesis were detected by agarose gel electrophoresis. (**E**) The results of DNA sequencing for the mutants of *TBP* gene deletion, insertion and point substitution. The left panels are the DNA sequencing maps for the mutants of TBP deletion, insertion and point substitution. The deletion position is marked with a red triangle (top), whereas the insertion and point mutations were framed by the red rectangle lines (middle and bottom), respectively. The right panels are the DNA fragments from the sequence maps (right panel), where the amino acids flanking each mutation site are indicated. The original and mutated bases were displayed in green and red colours, respectively.

Since the SMLP method can be repeatedly used for mutagenesis of large plasmids, we reasoned that this method can also be applied to the generation of gene mutants for small plasmids. We next constructed a pET-30a-TBP plasmid (about 6.3 kb) and designed the primer pairs for the generation of deletion, insertion, and point mutations (Fig. 9A, Table S4). Site-directed mutagenesis was performed using this plasmid as DNA templates and the method described in Fig. 2. Figure 9B-E shows that all of the TPB mutants, including TBP deletion, insertion and point substitution mutations, have been successfully generated by the SMLP method. Taken together, these data indicate that the SMLP method can be applied to the generation of substitution, deletion and insertion mutations for both large and small plasmids.

### The SMLP method can be applied to site-directed mutagenesis via three-fragment assembly

The recombinational ligation has been used for the assembly of over two fragments and site-directed mutagenesis. For instance, the Gibson Assembly Cloning kits from a commercial company (Synthetic Genomics and others) can be used for the assembly of 2–5 fragments. We next tested if the SMLP method could be applied to site-directed mutagenesis through the three-fragment assembly. The pLV-FLNA plasmid (17.3 kb) acted as DNA templates in the PCR reactions for three fragments, and three pairs of overlap PCR primers were designed as indicated in Fig. S2. PCR reactions were performed, and their products were detected by agarose gel electrophoresis. Data confirmed that three DNA fragments were obtained through PCR reactions (Fig. S3A-C). After purification, equal moles of these fragments were assembled in a 20 μL reaction mixture for 25 min under Exnase II, where the DNA quantity for any fragments was over 20 ng. The transformation was performed using 3 μL ligation samples, and all of the transformation mixtures were spread on an LB plate. Fig. S3D shows that 9 colonies were observed in the LB plate, indicating that three-fragment assembly could produce colonies, but it drastically reduced the number of colonies when compared with the two-fragment assembly (Fig. 6C). Whether these colonies contained correct plasmids was still unclear. Thus, we extracted the plasmids from these colonies using a Plasmid Miniprep kit (CWBiol), and the resulting plasmids were subjected to agarose gel electrophoresis and DNA sequencing. Agarose gel electrophoresis showed that only 3 in 9 plasmids (33.3%) were correct in size when compared to the original plasmid (Fig. S3E). DNA sequencing confirmed that three mutation sites within the correct plasmids were achieved (Fig. S3F). These data indicate that the SMLP method can be applied to site-directed mutagenesis through the three-fragment assembly, but the rate of the positive plasmids is greatly reduced when compared to the SMLP method with the two-fragment assembly.

## Discussion

Mutagenesis of large plasmids in vitro is a difficult task in the laboratories worldwide. Although a couple of methods have been developed for the mutagenesis of large plasmids^19,20^, the drawbacks for them are either very low efficiency for PCR or labour-consuming^20^. Moreover, we couldn’t generate any mutant from the pcDNA-FLNA plasmid using one of these two methods^19^ in our initial work. In this study, we developed a PCR-based method for site-directed mutagenesis of large plasmids (SMLP). We show that the SMLP method is highly efficient in site-directed mutagenesis and has a greater advantage than the conventional methods tested in this study. Apart from the high efficiency in mutagenesis, the SMLP method is simple because it includes only two PCR reactions and a fast recombinational ligation except for a super fidelity DNA polymerase. The mutagenesis of a gene using this method can be finished within 10 h (Table S5). Although the SMLP method takes a similar time to the other two conventional methods (Fig. 1B and C) when generating a gene mutant, the total time spent on mutagenesis for the SMLP method is less than these two conventional methods as the efficiency of PCR using a pair of complementary or partial complementary primers is usually very low due to the formation of primer-dimers (Fig. 7). In addition, the mutagenesis of large plasmids by the conventional methods hardly succeeds in the laboratories (Table S1), and the protocols must be constantly modified to obtain mutants. Thus, these two conventional methods likely consume more time than the SMLP method during the mutagenesis of a gene. In the SMLP method, however, PCR reactions are simplified because a long PCR reaction for large plasmids is divided into two short PCR reactions (PCR I and PCR II), which produce two linear DNA fragments. In addition, the SMLP method uses very little DNA as DNA templates so that Dpn I digestion is optional. The site-directed mutagenesis based on the PCR with a pair of inverse primers is relatively complicated; it includes PCR, phosphorylation, ligation with T4 DNA ligase, and transformation^12,13^. Therefore, this method consumes more time and labour when compared with the SMLP method (Table S5). Taken together, the SMLP method is not only highly efficient and but also time-saving when utilized for mutagenesis of large plasmids in vitro.

We show that alteration of the distance between two pairs of primers did not affect the efficacy of mutagenesis (Fig. 5), suggesting that the distance between two pairs of primers designed for the SMLP method may vary at any known sequence within a plasmid (Fig. S1 and Fig. 2A). The flexibility in designing primer pairs, especially for MAFP and MARP, would greatly enhance the chances of success for both PCR and mutagenesis. Furthermore, an extra function for the MAFP and MAFP primers is that they can be changed into a pair of mutation primers at any time (Fig. S2 and 3). This extra function of the MAFP and MAFP primers can accelerate the progress for the experiments of site-directed mutagenesis especially when multiple sites within a gene are required to be mutated (Fig. S3).

The SMLP method requires two pairs of partially complementary primers (Fig. 2A), and each primer pairs need separating and used in two different PCR reactions (Fig. 2B). Thus, it is convenient to modify at the 5′ end of each primer, and such modification does not affect PCR reaction. Using this advantage, we have generated deletion and insertion mutations within the *Flna* gene cloned in the large plasmid through the SMLP method (Fig. 8). These techniques have successfully generated gene mutants when a T5 5′–3′ exonuclease was utilized in the protocol^21^. Heydenreich et al. have established a high-throughput mutagenesis pipeline using two separate PCR reactions and a Gibson Assembly Cloning kit^22^. However, these studies are a vector-based cloning method and can only generate the mutants from the small plasmids. We have also noticed that the mutagenesis of the large plasmids could be completed using the Gibson Assembly Site-Directed Mutagenesis Kit (Synthetic Genomics or other commercial co.). However, it needs taking multiple steps to amplify DNA fragments to assemble a large plasmid because the manual from the kit recommends amplifying a fragment less than 5.5 kb for one PCR reaction; furthermore, the whole sequence for a plasmid must be known before mutagenesis as multiple pairs of overlap primers have to be designed within the plasmid. Thus, the mutagenesis of large plasmids by this method is complicated. In addition, the Gibson Assembly Site-Directed Mutagenesis Kit is very expensive for many laboratories. Although we have successfully generated several mutants from the large plasmids up to 17.3 kb using the SMLP method in this work, this method may be used for sit-directed mutagenesis for the plasmids over 17.3 kb as the assembly of three fragments has been confirmed successful by the SMLP method (Fig. S2 and 3). We show that the SMLP method can also complete the mutagenesis for small-sized plasmids (Fig. 9). Thus, the SMLP method can be applied to the generation of deletion, insertion, and point mutations for both large and small plasmids.

## Conclusions

In this study, we have developed a PCR-based method for site-directed mutagenesis of large plasmids (SMLP). In the SMLP method, we take advantage of several advanced techniques, including a high-efficiency DNA polymerase for large DNA fragment amplification, and two separate PCR reactions, and the recombinational ligation with a 5′–3′ exonuclease. These techniques were combined to form a high-efficiency protocol for the mutagenesis of large plasmids. This method exhibits excellent reproducibility when it is utilized to generate mutants for the plasmids up to 17.3 kb. We show that the SMLP method can be applied to the generation of point substitution, deletion, and insertion mutations for both small and large plasmids, and it has been confirmed successful for site-directed mutagenesis through three-fragment assembly. This method is simple, low-cost, and especially suitable for the laboratories that require the mutagenesis of a gene cloned in a large plasmid.

## Methods

### Plasmids, cells, and reagents

Filamin A gene (*Flna*, 7946 bp) was cloned into the pcDNA3.1 plasmid (Thermo Scientific) and the pLV-CMV-EGFP plasmid (Inovogen, Beijing, China). The human *TBP* gene was cloned into the pET-30a ( +) plasmid. *Escherichia coli* competent cells (DH5α) and DNA markers were purchased from Tsingke Biotech Co. (Wuhan, China). 2*Phanta Max master mix and Exnase II were purchased Vazyme Biotech LTD (Nanjing, China). Both Q5 high fidelity of DNA polymerase and *Dpn* I restriction enzyme were obtained from New England Biolab. Chemical reagents were obtained from the Sinopharm Chemical Reagent Co.

### Primers and PCR

The PCR primers used in this study were synthesized by Sangon (Shanghai) and dissolved in deionized distilled H_2_O. PCR was performed using 0.25 ng plasmids as DNA templates in a 40 μL PCR reaction mixture containing 20 μL Phanta Max master mix (2×) and 1 μL 25 μM forward and reverse primers. The thermal cycle program for PCR consisted of 32 cycles, and each cycle comprised 95℃ 1 min, 56℃ 30 s, 72℃ for 1–7 min, where the extension time at 72℃ depended on the size of DNA. PCR products were detected by agarose gel electrophoresis.

### PCR product purification, recombinational ligation, and transformation

Forty μL PCR products were purified using a DNA gel extraction kit (Axygen) according to the manufacturer’s manual. Thirty μL of the eluates were obtained from one PCR reaction, 3 μL of them were detected by agarose gel electrophoresis. The concentration of PCR DNA was measured using a Nanophotometer (Thermo Scientific). To complete DNA recombinational ligation in vitro, equal moles of DNA fragments from two independent PCR reactions (PCR I and PCR II) were mixed in a 20 μL reaction mixture containing 1 μL Exnase II ( a type of 5′ → 3′ exonucleases that can make overhang ends for double-strand DNA fragments) and 4 μL 5*CE II buffer. Typically, in this work, we mixed 30 ng of a 3.4-kb DNA fragment (a fragment derived from the primer pair of pLVFP/D1159ARP) with 120 ng of a 13.9-kb DNA fragment (a fragment derived from the primer pair of pLVRP/ D1159AFP) in 20 μL reaction mixture. Recombinational ligation was performed by incubating the reaction mixture at 37℃ for 20 min in a water bath. Three μL ligation samples were added into 45 μL *E. coli* (DH5α) competent cells obtained from Tsingke Biotech Co. (Wuhan, China) and incubated for 45 min on ice. After that, the competent cells were placed in a water bath at 42℃ and incubated for 90 s, followed by adding 200 μL SOC solution (0.5% Yeast Extract; 2% Tryptone; 10 mM NaCl; 2.5 mM KCl; 10 mM MgCl2; 20 mM Glucose) into the competent cells and incubating 45 min in a shaker at 37℃. When incubation finished, all of the SOC and competent cell mixture was spread in an LB plate containing the ampicillin with the final concentration of 50 μg/mL and incubated at 37℃ overnight in a bacterial incubator.

### Plasmids preparation and DNA sequencing

The individual colonies on the LB plates were inoculated using the LB medium containing the ampicillin with the final concentration of 50 μg/mL, where 3 mL of the LB medium were inoculated for each colony. The LB medium inoculated were cultured at 37℃ overnight in an incubator with vivid shaking. After incubating 16 hours, bacterial cells were harvested for plasmid preparation using a Plasmid Miniprep kit (CWBiol, China) according to the manufacturer’s manual, where 50 μL of DNA eluates were obtained. Three μL of the resulting plasmids were directly monitored by agarose gel electrophoresis in which the same amount of the original plasmids was loaded as a control. The plasmids with the correct size were further verified by DNA sequencing. The DNA sequencing was performed by Sangon Co. (Shanghai, China).

### DNA digestion, phosphorylation, and ligation

For site-directed mutagenesis based on PCR using a pair of complementary or partially complementary primers, DNA templates have to be digested with a restriction enzyme before transformation assays. Briefly, PCR was performed in a 25 μL reaction mixture containing 12.5 μL PCR master mix (Thermo Scientific/NEB/Vazyme Co), 200 ng plasmids and 1 μL 25 μM forward and reverse primers. When PCR was finished, PCR products were digested for 1 h under 10 U Dpn I at 37℃. A transformation assay was performed using 3 μL digestion samples as described for the SMLP method. For the site-directed mutagenesis based on PCR using a pair of inverse primers, PCR was performed in a 25 μL reaction mixture containing 12.5 μL PCR master mix (Thermo Scientific /NEB /Vazyme Co), 0.5 ng plasmids and 1 μL 25 μM forward and reverse primers. PCR products were purified with a DNA purification kit (CWBiol) and were then phosphorylated using T4 polynucleotide kinase (NEB) according to the manufacturer’s manual. DNA ligation was performed in a 20 μL reaction mixture containing 5 μL phosphorylated samples and 5 U T4 DNA ligase (Thermo Scientific). A transformation assay was performed using 3 μL ligation samples as described for the SMLP method.

### Statistical analysis for the rate of positive clones

Thirty of the colonies picked from the LB plate derived from the transformation with 3 μL ligation samples were inoculated with LB medium and cultured overnight at 37℃ in an incubator with vivid shaking. Plasmids were prepared using the plasmid Miniprep kit as described above, and their sizes were detected by agarose gel electrophoresis. The plasmids with the correct size were sent for DNA sequencing. The rate of the positive clones was obtained by calculating the percentage for the number of the mutants in the number of colonies inoculated. The data from three independent experiments were subjected to statistical analysis.

## Supplementary Information

Supplementary Information.
