# Supplementary material for: A high-efficiency method for site-directed mutagenesis of large plasmids based on large DNA fragment amplification and recombinational ligation
Source: Sci Rep. 2021 May 17;11:10454. doi: 10.1038/s41598-021-89884-z (PMC8129136; doi:10.1038/s41598-021-89884-z)
Supplement: Supplementary file 1 — Supplementary Information. [file 41598_2021_89884_MOESM1_ESM.pdf]

A high-efficiency method for site-directed mutagenesis of large plasmids based on large DNA fragment amplification and recombinational ligation

Kewei Zhang[1#], Xiaomei Yin[1#], Kaituo Shi[1#], Shihua Zhang, Juan Wang[2]， Shasha Zhao[1], Huan Deng[1], Cheng Zhang[1], Zihui Wu[1], Yuan Li[1], Xiangyu Zhou[1], Wensheng Deng[1]*

1. College of Life Science and Health, Wuhan University of Science and Technology

2. College of Materials and Metallurgy, Wuhan University of Science and Technology

#, these authors contributed this work equally

*, To whom correspondence may be addressed: Tel. 008618571542248, dengwensheng@wust.edu.cn

Fig. S1

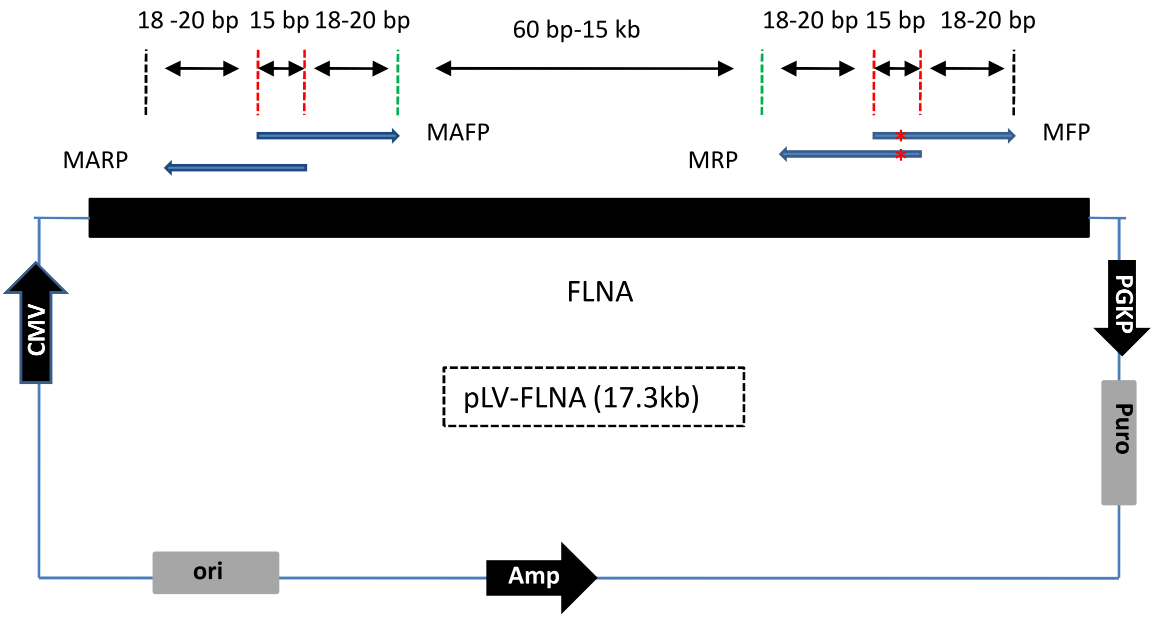

Fig. S1. A scheme showing the primer design principles for the SMLP method. The distance between two red dot lines represents the overlapping region for the primer pairs of MFP/MRP or MAFP/MARP (15 bp). The distance between the red dot line and the black or green dot line represents the length of primer without overlap (18-20 bp). The arrow line between two green dot lines represents the distance between the primer MAFP and the primer MRP, where the distance varies from 60 bp to 15 kb. The red stars in both MFP and MRP represent mutation sites. MFP, mutation forward primer; MRP, mutation reverse primer; MAFP, mutation assisting forward primer; MARP, mutation assisting reverse primer.

Fig. S2

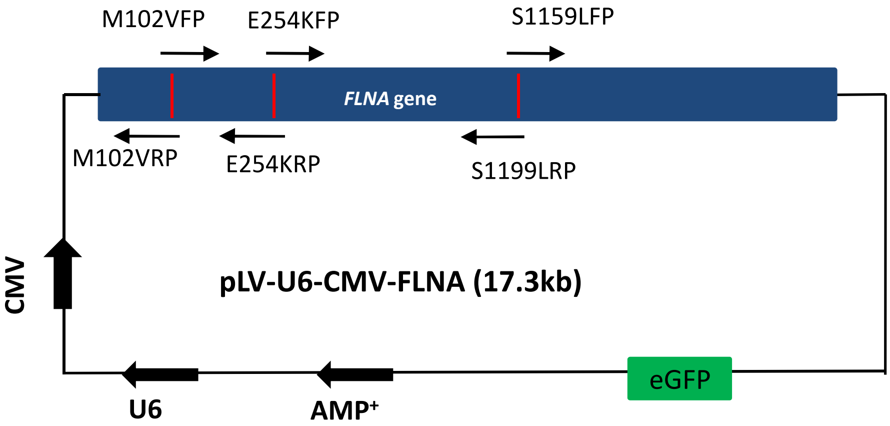

PCR I: M102VFP/E254RP; products, 450bp.

PCR II: E254FP/S119LRP; products, 2.835bp

PCR III: S1159FP/M102VRP; products, 14 kb

Fig. S2. **A scheme showing the locations of the primers on the pLV-FLNA plasmid designed for the assembly of three fragments**. Three pairs of mutated primers were used in three independent PCR reactions (PCR I, II, and III), which were performed using the primer pair as indicated (the bottom panel).

Fig. S3

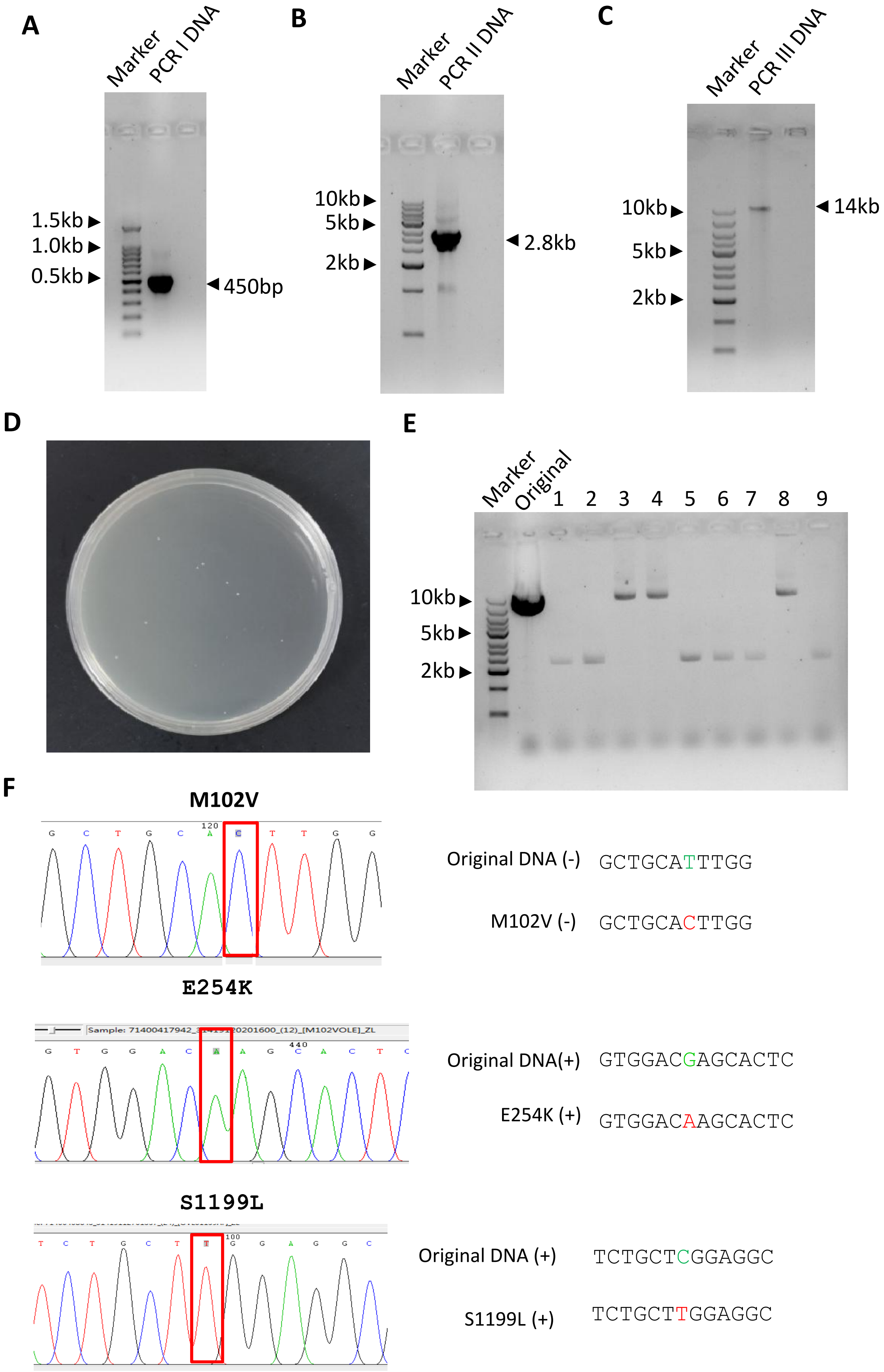
A
Marker
PCR I DNA
1.5kb
1.0kb
0.5kb
450bp
B
Marker
PCR II DNA
10kb
5kb
2kb
2.8kb
C
Marker
PCR III DNA
10kb
5kb
2kb
14kb
D
E
Marker
Original
1 2 3 4 5 6 7 8 9
10kb
5kb
2kb
F
M102V
Original DNA (-) GCTGCATTTGG
M102V (-) GCTGCACTTGG
E254K
Original DNA(+) GTGGACGAGCACTC
E254K (+) GTGGACAAGCACTC
S1199L
Original DNA (+) TCTGCTCGGAGGC
S1199L (+) TCTGCTTGGAGGC

Fig. S3. The SMLP can be applied to the assembly of three fragments based on large DNA fragment amplification. A) Detection of PCR I products by agarose gel electrophoresis; B) Detection of PCR II products by agarose gel electrophoresis: C) Detection of PCR III products by agarose gel electrophoresis. The products from PCR I, II, and III were obtained by PCR with the pLV-FLNA plasmids and the primers as indicated Fig. S2. D) A image for transformation results. The transformation was performed with 3 μL ligation samples, and all of the transformation samples were spread on a LB plate containing ampicillin with the final concentration of 50 μg/mL . E) Analysis of the plasmids derived from the mutagenesis based on the assembly of three fragments. F) The results of DNA sequencing for the plasmids with the correct size obtained in E. The left panels are the maps from the DNA sequencing for three mutated sites within the pLV-FLNA mutants. The right panel are the original and mutated sequence surrounding each mutated site.

Table S1. The PCR primer sequences and the results of PCR and mutagenesis from the conventional methods tested in this study

Methods based on PCR types	Primer name	Primer sequence	PCR results	No. of mutants
PCR and overlap extension PCR	FLNAClnF	GCGC**AAGCTT**ATGAGTAGCTCCCACTCTCGG	overlap extension PCR failed	0
	FLNAClnR	GCGCTCTAGATCAGGGCACCACAACGCGGTAG		
	M102VFP	TTCCGCCAAGTGCAGCTTGAG		
	M102VRP	CTCAAGCTGCA**C**TTGGCGGAA		
PCR with a pair of complementary primers	M102VCFP	GCGGCCCACTTTCCGCCAA**G**TGCAGCTTGAGAACGTGTCGGTG	failed	0
	M102VCRP	CACCGACACGTTCTCAAGCTGCA**C**TTGGCGGAAAGTGGGCCGC		
PCR with a pair of partially complementary primers	M102VPCFP	CGCCAA**G**TGCAGCTTGAGAACGTGTCGGTG	failed	0
	M102VPCRP	AGCTGCA**C**TTGGCGGAAAGTGGGCCGC		
PCR with a pair of inverse pimers	M102VIP-F	**G**TGCAGCTTGAGAACGTGTCGGTG	failed	0
	M102VIP-R	**C**TTGGCGGAAAGTGGGCCGC		

The red bases in the table represent restriction sites, and the blue bases are mutated bases. M102VFP, M102V forward primer; M102VRP, M102V reverse primer; M102VCFP, M102V complementary forward primer ; M102VCRP, M102V complementary reverse primer; M102VPCFP, M102V partially complementary forward primer; M102VPCRP, M102V partially complementary reverse primer; M102VIP-F, M102V inverse primer -forward; M102VIP-R, M102V inverse primer-reverse.

Table S2. The primers used for PCR reactions in the SMLP method

**Primer names**	**Primer sequences**	**Original codons/bases**	**Mutated codons/bases**
pcDNAFP	GGTACCGAGCTCGGATCCAC	N/A	N/A
pcDNARP	CCGAGCTCGGTACCAAGCTT	N/A	N/A
M102VFP	TCCGCCAA**GTG**CAGCTTGAGAACGTG	ATG (M)	GTG (V)
M102VRP	CAAGCTG**CAC**TTGGCGGAAAGTGGGCC	CAT	CAC
S149FFP	TGCACTAC**TTC**ATCTCCATGCCCATGTG	TCC (S)	TTC (F)
S149FRP	TGGAGAT**GAA**GTAGTGCAGGATCAGGG	GGA	GAA
P207LFP	GCTGTGCC**CTG**GGCCTGTGTCCTGACTG	CCG (P)	CTG (L)
P207LRP	ACAGGCC**CAG**GGCACAGCTGTCCACCAG	CGG	CAG
E254KFP	ACGTGGAC**AAG**CACTCTGTCATGACCTAC	GAG (E)	AAG (K)
E254KRP	AGAGTG**CTT**GTCCACGTTGGGGTCCAC	CTC	CTT
A1188TFP	GCTCGAGC**ACG**GGCAGCGCGGAGCTG	GCG (A)	ACG (T)
A1188TRP	CTGCC**CGT**GCTCGAGCAGTCCACTTG	CGC	CGT
S1199LFP	AGATCTGC**TTG**GAGGCGGGGCTTCCGGCC	TCG (S)	TTG (L)
S1199LRP	CCGCCTC**CAA**GCAGATCTCAATGGTCAG	CGA	CAA
pLVFP	ATGTACCCATACGATGTTCC	N/A	N/A
pLVRP	TCGTATGGGTACATTCTAGA	N/A	N/A
D1159AFP	TGCTTT**GCC**GCATCCAAAGTCAAG	GAC (D)	GCC (A)
D1159ARP	GGATGC**GGC**AAAGCAGGGAAC	GTC	GGC

Table S3. The primers used for generation of the pcDNA-FLNA-D1159A mutant by the SMLP method and the conventional methods tested in this study

Methods based on PCR types	Primer names	Primer sequences	The results of PCR and transformation
PCR I and PCR II(SMLP method)	pcDNAFP	GGTACCGAGCTCGGATCCAC	Specific bands were observed for PCR I and PCR II. Over a hundred of colonies were obtained
	pcDNARP	CCGAGCTCGGTACCAAGCTT	
	D1159AFP	TGCTTT**GCC**GCATCCAAAGTCAAG {GAC(D) →GCC(A)}	
	D1159ARP	GGATGC**GGC**AAAGCAGGGAAC	
PCR with a pair of complementary primers	D1159ACFP	CGTGGTTCCCTGCTTT**GCC**GCATCCAAAGTCAAGTG	No specific band was observed and no colony was obtained
	D1159ACRP	CACTTGACTTTGGATGC**GGC**AAAGCAGGGAACCACG	
	A1188TCFP	CAAGTGGACTGCTCGAGC**ACG**GGCAGCGCGGAGCTGAC	No specific band was observed and no colony was obtained
	A1188TCRP	GTCAGCTCCGCGCTGCC**CGT**GCTCGAGCAGTCCACTTG	
	S1199LCFP	GACCATTGAGATCTGC**TTG**GAGGCGGGGCTTCCGGCCG	No specific band was observed and no colony was obtained
	S1199LCRP	GGAAGCCCCGCCTC**CAA**GCAGATCTCAATGGTCAGC	
PCR with a pair of partially complementary primers	D1159APCFP	CTGCTTT**GCC**GCATCCAAAGTCAAGTG	No specific band was observed and no colony was obtained
	D1159APCRP	GGATGC**GGC**AAAGCAGGGAACGC	
	A1188TPCFP	TCGAGC**ACG**GGCAGCGCGGAGCTGAC	No specific band was observed and no colony was obtained
	A1188TPCRP	GCC**CGT**GCTCGAGCAGTCCACTTG	
	S1199LPCFP	ATCTGC**TTG**GAGGCGGGGCTTCCGGCCG	No specific band was observed and no colony was obtained
	S1199LPCRP	GCCTC**CAA**GCAGATCTCAATGGTCAGC	

Table S3 (continue)

PCR with a pair of inverse primers	D1159AIP-F	**CCGCATCCAAAGTCAAGTGC TCAG**	No specific band was observed and no colony was obtained
	D1159AIP-R	**G**CAAAGCAGGGAACCACGT GGGC	
	A1188TIP-F	**ACG**GGCAGCGCGGAGCTGA C	No specific band was observed and no colony was obtained
	A1188TIP-R	**T**GCTCGAGCAGTCCACTTG	
	S1199LIP-F	**TG**GAGGCGGGGCTTCCGGC CG	No specific band was observed and no colony was obtained
	S1199LIP-R	**AA**GCAGATCTCAATGGTCAG C	

The blue bases represent the mutated bases, the green base is the original base. pcDNAFP, pcDNA plasmid forward primer; pcDNARP, pcDNA plasmid reverse primD1159AFP, D1159A forward primer; D1159ARP, D1159A reverse primer; D1159ACFP, D1159A complementary forward primer ; D1159ACRP, D1159A complementary reverse primer; D1159APCFP, D1159A partially complementary forward primer; D1159APCRP, D1159A partially complementary reverse primer; D1159AIFP, D1159A inverse primer-forward; D1159AIRP, D1159A inverse primer-reverse. Other primers were designated with the same way as for D1159A.

Table S4. The primers used for the generation of substitution, deletion, and insertion by the SMLP method

Primer names	Primer sequences	Substitution/deletion/insertion
A1188delFP	CTCGAGCGGCAGCGCGGAGCTGACCATTG	GCG (A1188 deletion)
A1188delRP	CGCTGCCGCTCGAGCAGTCCACTTGG	GCG (A1188 deletion)
SCInsFP (SC, stop codon)	CGAGCGCGTAAGGCAGCGCGGAGCTGACCATTG	TAA insertion
SCInsRP (SC, stop codon)	CGCTGCCTTACGCGCTCGAGCAGTCCACTTGG	TTA insertion
TBPDFP	GAAGAGCAACAAAGGGCAGTGGCAGCTGCAGCCG	114bp (aa58-aa95) deletion
TBPDRP	CCCTTTGTTGCTCTTCCAAAATAG	114bp (aa58-aa95) deletion
TBPINFP	CAACAAAGGCAGGCAGTGGCAGCTGCAGCCG	CAG insertion
TBPINRP	CCTGCCTTTGTTGCTCTTCCAAAATAG	CTG insertion
TBPPMFP	CAGCAACAGGCAGTGTCAGCTGCAGCCGTTCAGC	GCA → TCA
TBPPMRP	GCTGAACGGCTGCAGCTGACACTGCCTGTTGCTG	TGC → TGA
TBPMAFP	AAAGGGATTCAGGAAGACGACG	N/A
TBPPMRP	TCCTGAATCCCTTTAGAATAGG	N/A

Table S5. Comparison of the time consumed for generation of the gene mutants for a 13-kb plasmid between the SMLP method and the conventional methods

Methods for mutagenesis	Procedures and the time consumed in each step	Total time
The SMLP method	PCR I and II including preparation work , 4.5 hours; DNA detection and purification, 1.5 hours; recombinational ligation, 0.5 hours; transformation, 2 hours.	8.5 hours
The method based on PCR with a pair of complementary primers	PCR including preparation work, 5.5 hours; Dpn I digestion,1.5 hour; transformation, 2 hours.	8.0 hours
The method based on PCR with a pair of partially complementary primers	PCR including preparation work , 5.5 hours; Dpn I digestion,1.5 hour; transformation, 2 hours.<br>Or the procedures for recombinational ligation method:<br>PCR including preparation , 5.5 hours;<br>Dpn I digestion,1.5 hour; DNA detection and purification, 1.5 hours;<br>Recombinational ligation, 0.5 hours;<br>transformation, 2 hours.	8.0 hours or 10 hours
The method based on PCR with a pair of inverse primers	PCR including preparation work , 5.5 hours; DNA detection and purification, 1.5 hours; phosphorylation, 1 hour; ligation, over 2 hours; transformation, 2 hours	12 hours at least
The method based on PCR, overlap extension PCR and subcloning	PCR and detection, 4 hours, overlap extension PCR and detection, 4 hours, subcloning (digestion, ligation and transformation) 1-2 days	2 days at least

Note that all of methods were performed with Phanta Max super-fidelity DNA polymerase, which can synthesize at least 1 kb per 30 seconds (it can reach up 1 kb per 10 seconds according to the manufacturer's manual). PCR time for full plasmid (13 kb) was assessed based on 32-cycle program, each cycle takes 10 min (for example, 95℃ 1 min, 56 ℃ 0.5 min, 72 ℃ 7 min and the time consumed for temperature change). PCR for full plasmids usually takes longer time than the PCR in the SMLP method because the PCR has been divided into two short PCR reactions in the SMLP method.
